# Rates of inclusion of teenagers and young adults in England into National Cancer Research Network clinical trials: Report from the National Cancer Research Institute (NCRI) Teenage and Young Adult Clinical Studies Development Group

**DOI:** 10.1038/sj.bjc.6604751

**Published:** 2008-11-25

**Authors:** L Fern, S Davies, T Eden, R Feltbower, R Grant, M Hawkins, I Lewis, E Loucaides, C Rowntree, S Stenning, J Whelan

**Affiliations:** 1 Department of Oncology, University College London Hospitals NHS trust, London NW1 2PG, UK; 2 Teenage Cancer Trust, 3rd Floor, 93 Newman Street, London W1T 3 EZ, UK; 3 Academic Unit of Adolescent and Paediatric Oncology, c/o Teenage Cancer Trust Young Oncology Unit, Christie Hospital NHS Trust, Withington, Manchester M2 4 BX, UK; 4 Paediatric Epidemiology Group, Centre for Epidemiology & Biostatistics, University of Leeds, Leeds LS2 9LN, UK; 5 St Brycedale Surgery, St Brycedale Road, Kirkcaldy, Fife KY1 1PH, UK; 6 Department of Public Health and Epidemiology, University of Birmingham, B15 2TT, UK; 7 University of Leeds, Leeds LS2 9LN, UK; 8 National Cancer Research Institute, PO Box 123, Lincoln Inn Fields, London WC2A 3PX, UK; 9 Department of Haematology, University Hospital of Wales, Heath Park, Cardiff CF14 4XN, UK; 10 Medical Research Council Clinical Trials Unit, 222 Euston Road, London NW1 2DA, UK

**Keywords:** teenagers and young adults, clinical trials, accrual rates, age inequalities in cancer care

## Abstract

Poor inclusion rates into clinical trials for teenagers and young adults (TYA; aged 13–24 years) have been assumed but not systematically investigated in England. We analysed accrual rates (AR) from 1 April 2005 up to 31 March 2007 to National Cancer Research Network (NCRN) Phase III trials for the commonest tumour types occurring in TYA and children: leukaemia, lymphoma, brain and central nervous system, bone sarcomas and male germ cell tumours. AR for 2005–2007 were 43.2% for patients aged 10–14 years, 25.2% for patients aged 15–19 years, and 13.1% for patients aged 20–24 years in the tumour types analysed. Compared with accrual from 1 April 2005 to 31 March 2006, AR between 1 April 2006 and 31 March 2007 increased for those aged 10–14 and 15–19 years, but fell for those aged 20–24 years. AR varied considerably among cancer types. Despite four trials being available, patients over 16 years with central nervous system tumours were not recruited. Rates of participation in clinical trials in England from 2005 to 2007 were much lower for TYA older than 15 years compared with children and younger teenagers. The variations in open trials, trial age eligibility criteria and extent of trial activation in treatment centres in part explain this observation. Other possible influences, such as difficulties associated with the consent of TYA require further evaluation. Closer dialogue between those involved in planning and running trials for children and for adults is necessary to improve trial availability and recruitment. Further research is required to identify trends in trial availability and accrual for those tumours constituting the remaining 26% of TYA cancers.

The excellent rates of survival now commonplace for children affected by cancer have been in part attributed to very high rates of accrual to clinical trials at diagnosis (Stiller and Eatock, 1999). It has been estimated that as many as 70% of children in the Western World will enter a clinical trial (Ablett and Pinkerton, 2003). Inclusion in clinical trials is widely regarded as being associated with enhanced quality-of-care, the attention of a broader group of specialised professionals (Ferrari and Bleyer, 2007), and is seen as an indicator of an optimum standard of care. Recent data from the United States have documented a rapid fall-off after the age of 14 years in the proportion of patients enroled in National Cancer Institute-supported trials and it is this same group that appears to have gained least from the overall improvements in survival from cancer experienced by younger children and by adults aged more than 40 years (Bleyer *et al*, 2005).

Poor inclusion rates into clinical trials for teenagers and young adults have been presumed by clinical investigators for some time but had not systematically investigated in England. We set out to determine current inclusion rates of those with the most common cancers occurring in the age range of 13–24 years into National Cancer Research Network-endorsed (NCRN) clinical trials between 1 April 2005 and 31 March 2007. We wished to compare this with similar data for children and investigate any factors, which may be associated with better trial enrolment.

## Methods

We analysed accrual by age to phase III, intervention trials recruiting newly diagnosed patients with selected tumour types under the auspices of the NCRN. Similarly, we analysed trials in selected tumour types from the Children's Cancer and Leukaemia Group (CCLG), some of which were not randomised. We included NCRN-funded studies and other studies approved for inclusion in the NCRN portfolio (http://public.ukcrn.org.uk/search/Portfolio.aspx?Level1=1).

We selected cancer subtypes for analysis based on the frequency of presentation within the following age groups: children (aged 0–12 years) and teenagers and young adults (TYA) (aged 13–24 years). In TYA, lymphomas, brain and central nervous system (CNS) tumours, germ cell tumours, leukaemia and bone sarcomas together make up 74% of the newly diagnosed patients (Alston *et al*, 2007). To compare accrual rates for children we then analysed the most common tumour types accounting for a similar proportion of newly diagnosed tumours in this cohort: leukaemia, lymphoma, brain and central nervous system tumours which account for 73% of tumours (Childhood Cancer Research Group, 2007). Trials in these tumour types were selected from the NCRN and CCLG portfolios. Only trials open for accrual at any time between 2005 and 2007 were included (NCRN: http://www.ncrn.org.uk). Trials endorsed by the NCRN collect data from 1 April to 31 March each year. We requested accrual data for the same time periods from CCLG.

We determined the clinical trial availability by manually searching each trial summary to select study type; study design; eligibility criteria and current recruitment status. The trials selected are shown in Table 1. We obtained accrual data for the selected trials by contacting the trial's principle investigator. Age at trial entry for included patients was most often released by the clinical trial co-ordinator, statistician or data manager. Patients were then grouped into quintiles corresponding to the 5-year age groups used by the Office for National Statistics (ONS; 0–4, 5–9, 10–14, 15–19, and so on). We obtained the ages of all patients recruited to the selected trials, not just those aged 13–24 years. We were thus also able to compare the inclusion rates of TYA in trials relevant to their age group with the inclusion rates of older adults to the same trials. The tumour types analysed account for just 12% of tumours occurring in those aged 25–59 years (Office for National Statistics, 2005).

We obtained cancer incidence data from the ONS in 2004, Series MB1 no. 35, which lists cancer registrations in England (Office for National Statistics, 2005). The following ICD-10 diagnosis codes were used: CNS C71, leukaemia C91–C95, Hodgkin's lymphoma C81, non-Hodgkin's lymphoma C82–C85, testis C62, sarcoma C40, C41. As this was the most recent incidence data available, we have assumed a similar number of incident cases during the two 12-month periods analysed, 2005–2006, and 2006–2007. The percentage accrual of patients, or accrual rate, was expressed as the proportion of patients entered onto the selected trials compared with the number of new cases diagnosed in 2004.

We applied simple descriptive statistics judging this appropriate for an observational dataset for which we could not control the sample size or number of incidence cases. In addition, statistical comparisons between years and groups could only be deemed valid if we were able to use real time incidence data rather than deriving information from the most recent years.

## Results

**Figure 1:**
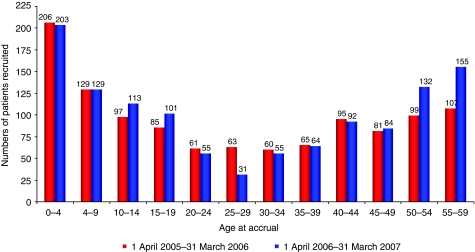
Number of newly diagnosed cancer patients entered in NCRN and CCLG lymphoma, leukaemia, CNS, bone sarcoma and male germ cell phase III trials, 1 April 2005–31 March 2007.

A total of 2608 cases of cancer (excluding non-melanoma of the skin) were recorded in patients aged 0–24 years in 2004. The number and proportion of the incident cases with cancer of the subtypes we selected were 1604 or 61.1% of cases. Figure 1 depicts the number of patients in England who were entered onto the selected trials between 2005 and 2007. A total of 1214 patients aged 0–59 years were enrolled onto trials for the selected tumour types during 2006–2007, representing 14.3% of 8501 new diagnoses between the ages of 0 and 59 years for these selected tumour types during 2004. This is higher than the NCRN figure of approximately 6.0% accrual to randomised trials (National Cancer Research Network, 2006) 2006–2007, and is due to the high accrual rates of paediatric patients through CCLG centres, compared with TYA and adult tumour types. Accrual rates for 2005–2006 in the selected tumour types were slightly lower at 13.5% (1148 patients).

**Figure 2:**
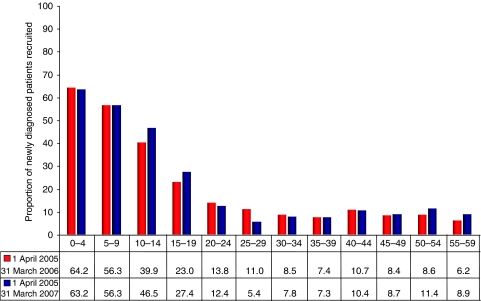
Proportion of newly diagnosed cancer patients entered in NCRN and CCLG lymphoma, leukaemia, CNS, bone sarcoma and male germ cell phase III trials, 1 April 2005–31 March 2007.

The decline in trial participation after 15 years is particularly notable when trial entry is shown as a proportion of newly diagnosed cases. This is prominent in both years studied (Figure 2). During 2005–2006 within the tumour types analysed, 39.9% of patients aged 10–14 years (97 of approximately 243 patients) were entered into clinical trials. This fell to 23.0% for 15–19-year-olds (85 of approximately 369 patients), and just 13.8% for patients aged 20–24 years (61 of approximately 442 patients). During 2006–2007 accrual to trials for patients aged 10–14 and 15–19 years improved by 6.6 and 4.4%, respectively. This was accompanied by a decline of 1.4% in accrual rates for patients aged 20–24 years.

**Figure 3:**
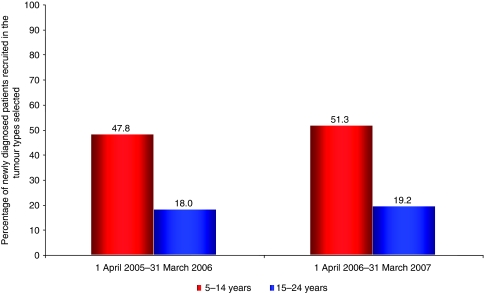
Proportion of newly diagnosed patients aged 5–14 and 15–24 years entered in NCRN and CCLG lymphoma, leukaemia, CNS, bone sarcoma and male germ cell phase III trials, 1 April 2005–31 March 2007.

We also divided the cohort into two 10-year age spans to broadly reflect care within children's or adult services. New cancers in the tumour types studied occurred almost twice as often in those aged 15–24 years (811 cases in the tumour types analysed) compared with 5 to 14-year-olds (472 cases in the tumour types analysed). During 2005–2006 the percentage of patients entering trials changed from 47.8% for ages 5–14 years (226 of approximately 472 patients), to just 18.0% of patients 15–24 years (156 of approximately 811 patients). Similarly, between 2006 and 2007 the percentage of patients aged 5–14 years entering trials was 51.3% (242 of approximately 472 patients), compared with 19.2% of patients aged 15–24 years (156 of approximately 811 patients; Figure 3). The average over both years shows that approximately 50% of patients aged between 5 and 14 years will be recruited to trials compared with less than 20% of patients aged 15–24 years for selected tumour types.

Accrual of TYA was lower than that for children in the following tumour types: leukaemia, CNS tumours, and bone sarcoma (Table 2). Accrual of TYA only exceeded that of children in male germ cell tumours (MGCT) where no patients under the age of 14 were recruited to trials during 2005–2007 reflecting the low incidence rate of this tumour in the 10–14 age group (seven new cases in 2004). Two trials were open for TYA with MGCT which have overlapping age eligibility criteria, CCLG GC3, a Phase III trial for a newly diagnosed extra cranial malignant germ cell tumour with an upper age limit of 18 and NCRN TE3, a Phase III trial for male patients with good risk metastatic germ cell cancer of the testis with age eligibility of 16–50 years. For 2005–2006 accrual for patients aged 20–24 years was just 2.3% (three patients of approximately 131) compared with 5.2% of patients aged 15–19 years (three patients out of approximately 58; one being recruited to the CCLG trial and two to the NCRN TE-3 trial).

Few trials were open during 2005–2007 for the commonest lymphomas affecting children and TYA. For example, there were no studies for patients aged 0–17 years with Hodgkin's lymphoma. Between 2005 and 2006, we found similar accrual of paediatric and TYA patients to lymphoma trials, 6.3% of patients aged 5–14 years *vs* 6.6% of patients aged 15–24 years (Table 2). However, analysis by quintiles demonstrated a pattern of decline in patients over 15 years (Table 2). Approximately 8.6% of patients aged 10–14 years (7 of approximately 81 patients) were recruited to trial between 2005 and 2006. Despite almost twice the incidence of lymphoma in patients aged 15–19 years, the accrual rates were almost half that of those aged 10–14 years, with just 4.5% of patients aged 15–19 years being recruited to trials (7 of approximately 156 patients). However, accrual rates increase again for patients aged 20–24 years with 8.3% of patients entering trials (16 of approximately 192 patients). During 2006 and 2007 accrual rates for 10–14 years and 20–24 years fell by 6.1 and 0.5%, respectively. Accrual for patients aged 15–19 years remained the same over both time periods.

**Figure 4:**
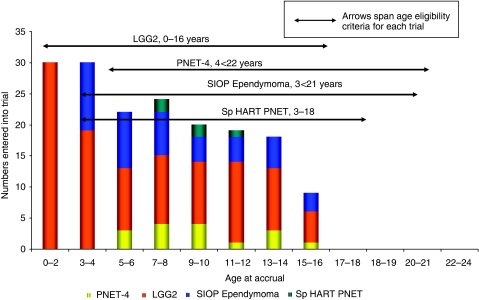
Number of newly diagnosed patients recruited to CCLG CNS trials from opening to March 2007. Age eligibility criteria for each trial are shown.

Brain and other CNS tumours account for approximately 14.3% of cancers in patients aged 13–24 years (Alston *et al*, 2007). Accrual of TYA to relevant trials for the 2-year period 2005–2007 was very low. Four CCLG trials were open recruiting newly diagnosed patients, the upper age eligibility of these trials ranging from 16 to 22 years. Accrual by age, in 2-year intervals is shown in Figure 4 together with trial availability and age eligibility criteria. No patients over 16 years had been included from opening these trials until 31 March 2007. There were no open trials during this period for patients with newly diagnosed CNS tumours aged 23–24 years.

For bone sarcomas there were two large international randomised trials open, one each for Ewing's tumours (EURO-Ewing's 99) and osteosarcoma (EURAMOS-1), which together account for over 90% of bone sarcomas in TYA. These trials have upper age limits of 50 and 40 years respectively. The first year of accrual data analysed between 1 April 2005 and 31 March 2006 included only 6 months of accrual data to EURAMOS 1, which opened in England in September 2005. Accrual to these two trials for patients aged 0–59 years was 4.2% for 2005–2006 and 24.1% for 2006–2007. A decline in accrual beyond the age of 15 still persists despite an age eligibility criterion, which spans the TYA age range. Between 2005 and 2006, accrual of 10 to 14-year-olds was 56.3% (nine of approximately 16 patients), and 22.2% for 15 to 19-year-olds (10 of approximately 45 patients). Between 2006 and 2007 almost 100% of patients aged 10–14 years were recruited, falling to 73.3% of patients aged 15–19 years.

The NCRN and CCLG leukaemia trials demonstrated high accrual across all age groups for 2005–2006, (Table 2). Accrual for patients aged 10–14 years was approximately 100%, falling to 91.5% for patients aged 15–19 years, with similar accrual rates (92.9%) for patients aged 20–24 years. However, for 2006–2007 this fell considerably across all age groups reflecting the closure of the main study for acute lymphoblastic leukaemia (ALL), UKALLXII, for a common subset of patients (Philadelphia negative ALL). During 2006–2007 accrual rates into NCRN and CCLG haematology portfolios fell from approximately 100% of patients aged 10–14 years, to just 77.5% of patients aged 15–19 years with a further decline for those aged 20–24 years to 48.2%, which was almost half the accrual rate compared with 2005–2006 for this age group.

## Discussion

Improving recruitment to high quality clinical trials has been a constant theme in strategies aimed to improve cancer outcomes, outlined in the NHS Cancer Plan of 2000 and perhaps more notably in the recent Cancer Reform Strategy 2007 which specifically refers to age inequalities in clinical trial accrual (Department of Health, 2000, 2007). Patients in trials, in addition to potentially receiving improved therapy, may experience a ‘secondary gain’, benefiting from enhanced quality-of-care and the attention of a broader group of specialised professionals (Ferrari and Bleyer, 2007). The excellent survival rates now achieved for many children with cancer relate in some part to high rates of inclusion in clinical trials. We set out to investigate the extent to which teenagers and young adults with frequently presenting tumour types were being recruited into clinical trials supported by the NCRN and CCLG. No single source of the data was available and we have developed a methodology which may be used to monitor changes in accrual in the future. We have shown that there is a substantial fall in the accrual of patients to trials beginning after the age of 14 for the tumour types analysed. This is consistent with data reported from the United States, Italy and Australia (Bleyer *et al*, 2005; O'Brien *et al*, 2006; Ferrari and Bleyer, 2007). The reasons are likely to be complex and multiple and require further investigation.

We have developed a methodology that allows up-to-date assessement of age-dependant trial accrual. We have demonstrated year by year sensitivity to changes in accrual resulting from trials opening and closing and have not been limited to trials emanating from a single trial organisation, but rather sought all those that may be relevant to TYA. Monitoring of interventions to improve trial accrual will now be possible in an accurate, and timely way. This approach contrasts with reports from other countries which have either described accrual rates from several years previously (Bleyer *et al*, 2005, 2006) or have not detailed the data sources and methodology (Ferrari and Bleyer, 2007).

Trials may not be open for all stages of all tumour types at all times. In the period studied, few if any, trials were available for some of the cancers that are common in young people, including Hodgkin's lymphoma and germ cell tumours. During 2005–2006, accrual to acute leukaemia trials was very high with little evidence of an age effect reflecting both broad eligibility criteria and a committed clinical community. However, this was affected by the closure of UKALL XII to Philadelphia negative ALL patients with a significant decrease in accrual across all ages during 2006–2007. Bone sarcoma trials also have age eligibility criteria spanning the biological spectrum of osteosarcoma and Ewing's sarcoma. Despite this, the accrual rate of 15 to 24-year-olds was approximately half that of 5 to 14-year-olds in both years analysed. In the UK most paediatric oncology services care for children up to the age of 16. The decreased accrual rate in 15 to 24-year-olds may be accounted for by an altered priority for clinical research in rare cancers in adult oncology services or a smaller proportion of older patients meeting other eligibility criteria. Further research is required to elucidate this.

The criteria which define the age range of patients eligible for trial entry are an important determinant of accrual. Commonly these reflect the source of a clinical trial, whether developed by paediatric or adult investigators rather than the age incidence of the particular tumour type. We found examples where age eligibility criteria overlapped between trials, presenting potential confusion for clinicians and patients. Emergence of data from the US, France, Holland, UK and now Sweden, have demonstrated that TYA with acute lymphoblastic leukaemia treated on paediatric protocols have better event-free survival and overall survival compared with protocols used for adults (Stock *et al*, 2000; Boissel *et al*, 2003; De Bont *et al*, 2004; Hallbook *et al*, 2006; Ramanujacher *et al*, 2007). These data resulted in co-operation within the UK between paediatric and adult haematologists. Consequently, the age eligiblity criteria of the current trial for ALL has been ammended from 18 to 20 and now to 25 years. This co-operation has resulted in additional accrual of TYA to this trial and may provide a model for other cancers.

Brain tumours are the most common cause of death from cancer in 15 to 24-year-olds (Geraci *et al*, 2007). A recent survival analysis by Birch *et al* (2008) has demonstrated there has been no sustained improvements for TYA with high-grade brain tumours during 1979–2003. Four trials were open with upper age limits ranging between 18 and 21 years during the period studied. No patients over 16 years with brain tumours have been included in these trials since opening. This clearly demonstrates that measures beyond the setting of age eligibility criteria appropriate to tumour biology are required to improve rates of inclusion of young people. This may include enhanced dialogue between research groups representing paediatric and adult services during the planning of new trials.

These data will serve as a benchmark for assessing the success of measures to improve clinical trial accrual of TYA across England. The analysis only reflects trials open to recruitment during 2005–2007. The effect of opening and closing trials is visible within this first 2-year analysis. Large trials such as the osteosarcoma study, EURAMOS-1, which did not start accruing in the UK until September 2005, made a significant improvement to the overall accrual rate across all ages. However, an age-dependent effect was still evident. The CCLG germ cell tumour study GC-3, which did not have all centres activated during 2005–2006, was also under-represented in the first year of analysis. For this trial, accrual did not appear to improve markedly as a consequence of activation of more centres.

We have used cancer incidence data from 2004 as this is most recently available, but is not concurrent with our accrual data which is between 1 April 2005 and 31 March 2006. Although it is unlikely that changes in incidence will have significantly affected the results, difficulties in the tumour classification used by cancer registries may have led to inappropriate inclusion of some tumours, for example some benign brain tumours. Trials may include patients with only certain stages of disease and we were unable to refine the incidence data to account for this, for example to accurately determine the proportion of patients with good risk GCT who would be potentially eligible. We also anticipated reporting accrual data by each National Cancer Research Network but accurate cancer incidence data in the required age bands was not available. Further, we encountered concerns about the release of small case numbers where the identification of individual patients is seen as a potential hazard.

The data we have presented covers approximately 74% of TYA tumours. Further study is required to identify trends in trial availability and accrual for those tumours constituting the remaining 26% of TYA cancers. We anticipate that trial participation will be lower for this cohort as the range of tumour types is greater including early onset of common adult carcinomas with smaller numbers of incident cases for each cancer.

TYA are less likely than children to be included in clinical trials of the most common cancers for their respective age groups. Improvements in accrual may contribute to improved treatment outcomes. This may be achieved through giving greater consideration to TYA in the planning of clinical trials and ensuring that relevant trials are active in centres treating young people. Particular attention is required to improve trial accrual in cancers where survival is poor, such as brain and bone tumours. Recognition of TYA in new performance incentives for cancer research networks may be an effective tool to improve access and accrual. A greater degree of centralisation of care for TYA is expected to result from the implementation of the recommendations of Improving Outcomes in Children and Young People with Cancer (National Institute for Clinical Excellence, 2005). This may overcome some of the organisational difficulties of maintaining broad trial portfolios in rare tumours. However, these data reflect a complex interplay of contributing factors including: the breadth of the national trial portfolio; variation in activation of trials between centres and networks; difficulties investigating rare cancers and changing attitudes to consent and participation among young people. Future research will involve identifying barriers to accrual and methods to overcome these so that TYA have equal access and accrual to clinical trials compared with their paediatric and adult counterparts.

## Figures and Tables

**Table 1:** Trials included in the analysis, including age eligibility criteria, and status during the reporting period, 1 April 2005–31 March 2007

**Portfolio/trial acronym**	**Title**	**Phase**	**Age eligibility criteria**	**Status during 1 April 2005–31 March 2007**
*Bone sarcoma*				
EURAMOS-1	A randomised trial of the European and American Osteosarcoma Study Group to optimise treatment strategies for resectable osteosarcoma based on histological response to pre-operative chemotherapy	III	40 years	Opened September 06
EUROEWINGS 99 (ET 2000 03)	European Ewing Tumour Working Initiatives of National Groups: Ewing Tumour Studies 1999	III	50 years	Open
				
*Brain and central nervous system*				
LGG 2004 (CNS 2004 03)	Cooperative multicentre study for children and adolescents with low-grade glioma	III	0–16 years	Open
PNET4 (CNS 2003 05)	A prospective randomised controlled trial of hyperfractionated *vs* conventionally fractionated radiotherapy in standard risk medulloblastoma.	III	4–22 years	Closed December 2006
SIOP Ependymoma (CNS 1999 04)	SIOP study of combined modality treatment in childhood ependymoma	III	3–21 years	Open
st PNET (CNS 2004 01)	Hyperfractionated Accelerated Radiotherapy (HART) with chemotherapy (Cisplatin, CCNU, Vincristine) for non-pineal supratentorial primitive neuroectodermal tumours	III	3–18 years	Open
				
*Germ cell (male)*				
BEP Continuous infusional bleomycin – TE3	A randomised phase III toxicity study of day 2,8,15 short (30 m ) *vs* day 1,2,3 long (72 hours) infusion bleomycin	III	16–50 years	Open
GC 3 (GC 2005 04)	Protocol for the treatment of extracranial germ cell tumours in children and adolescents	III	0–18 years	Opened May 2005
				
*Leukaemia*				
AML 15	Medical Research Council working parties on leukaemia in adults and children. Acute myeloid leukaemia trial 15	III	< 60 years	Open
EsPhALL	European Intergroup Study on post induction treatment of Philadelphia positive acute lymphoblastic leukaemia with imatinib	III	< 18 years	Open
MRC CLL5	The value of autografting younger patients with high risk chronic lymphocytic leukaemia (cll). A Randomised Phase III Intergroup Trial	III	>18 years	Open
SPIRIT	STI571 Prospective International Randomised Trial. A phase III, prospective randomised comparison of imatinib 400 mg *vs* imatinib 800 mg *vs* imatinib plus pegylated interferon in patients with newly-diagnosed chronic myeloid leukaemia.	III	>18 years	Open
UKALL XII	Medical Research Council Trial for adult patients with acute lymphoblastic leukaemia Under 56 years of age. To compare related donor transplant *vs* autologous transplant *vs* chemotherapy.	III	15–55 years	Closed to Ph negative patients December 2006
UKALL2003	Medical Research Council working party on leukaemia in Children. UK National Acute Lymphoblastic Leukaemia (ALL) Trial UKALL 2003	III	1–18 years	Open
				
*Lymphoma*				
ALCL 99 (NHL 2000 06)	International protocol for the treatment of childhood anaplastic large cell lymphoma	III	0–22 years	Open
BNLI MCD *vs* FMD	BNLI-Randomised Control Trial of MCD *vs* FMD in follicular NHL	III	18–70 years	Closed April 2006
Mantle Cell P3	A Randomised Controlled Trial of fludarabine/cyclophosphamide combination with or without rituximab in patients with untreated mantle cell lymphoma	III	>18 years	Opened December 2006
PRIMA	A multicentre, phase III, open-label, randomised study in patients with advanced follicular lymphoma evaluating the benefit of maintenance therapy with Rituximab (MabThera) after induction of response with chemotherapy plus Rituximab in comparison with no maintenance therapy	III	>18 years	Closed March 2007
R-CHOP 14 *vs* 21	A phase III multicentre Randomised Clinical Trial comparing rituximab with CHOP given every 14 days and rituximab with CHOP given every 21 days for the treatment of patients with newly diagnosed diffuse large B cell non-Hodgkin's lymphoma	III	>18 years	Open
BNLI STANFORD V	Protocol for a randomised phase III study of the Stanford V regimen, compared with ABVD for the treatment of advanced Hodgkin's disease	III	18–60 years	Closed March 2008^a^
Waldenstom's study	A randomised trial of Chlorambucil *vs* Fludarabine as initial therapy of Waldenström's Macroglobulinaemia and Splenic lymphoma with villous lymphocytes	III	>18 years	Open
Watch and Wait	Rituximab in treating patients with newly diagnosed Stage II, Stage III, or Stage IV Follicular Non-Hodgkin's Lymphoma	III	>18 years	Open

Abbreviations: BNLI: British National Lymphoma Investigation; CCNU: Lomustine; FMD: Fludarabine, Mitoxantrone and Dexamethasone; MCD: mitoxantrone, chlorambucil and dexamethasone; SIOP: International Society of Paediatric Oncology.

a This trial was suspended prior to closing.

**Table 2:** Percentage of patients entering into trial for TYA-specific tumours aged 0–24 years between 1 April 2005–31 March 2007

**Portfolio**	**1 April 2005–31 March 2006**	**1 April 2006–31 March 2007**	**% Change**
*Bone*			
0–4 years	0.0	66.6	+66.6
5–9 years	37.5	68.7	+31.2
10–14 years	56.3	100.0	+43.7
15–19 years	22.2	73.3	+51.1
20–24 years	28.6	42.8	+14.2
Children aged 5–14 years	46.9	100.0	+53.1
Older teenagers and young adults, 15–24 years	24.2	63.6	+39.4
			
*Brain and central nervous system*			
0–4 years	28.4	29.7	+1.3
5–9 years	20.0	32.9	+12.9
10–14 years	18.3	20.7	+2.4
15–19 years	17.9	10.3	−7.6
20–24 years	0.0	0.0	0.0
Children aged 5–14 years	19.1	26.3	+7.2
Older teenagers and young adults, 15–24 years	8.6	4.9	−3.7
			
*Germ cell*			
0–4 years	0.0	100.0	+100.0
5–9 years	0.0	0.0	0.0
10–14 years	0.0	0.0	0.0
15–19 years	5.2	3.4	−1.8
20–24 years	2.3	3.1	+0.8
Children aged 5–14 years	0.0	0.0	0.0
Older teenagers and young adults, 15–24 years	3.2	3.2	0.0
			
*Leukaemia*			
0–4 years	84.1	77.7	−6.4
5–9 years	99.1	85.5	−13.6
10–14 years	100.0	100.0	+0.0
15–19 years	91.5	77.5	−14.0
20–24 years	92.9	48.2	−44.7
Children aged 5–14 years	100.0	91.3	−8.7
Older teenagers and young adults, 15–24 years	92.3	64.5	−27.8
			
*Lymphoma*			
0–4 years	5.0	10.0	+5.0
5–9 years	0.0	3.2	+3.2
10–14 years	8.6	2.5	−6.1
15–19 years	4.5	4.5	0.0
20–24 years	8.3	7.8	−0.5
Children aged 5–14 years	6.3	2.6	+3.7
Older teenagers and young adults, 15–24 years	6.6	6.3	+0.3

## References

[bib1] Ablett S, Pinkerton CR, United Kingdom Children's Cancer Study Group (UKCCSG) (2003) Recruiting children into cancer trials – role of the United Kingdom Children's Cancer Study Group (UKCCSG). Br J Cancer 88: 1661–1665 12771976 10.1038/sj.bjc.6600990 PMC2377132

[bib2] Alston RD, Rowan S, Eden TOB, Moran A, Birch JM (2007) Cancer incidence patterns by region and socio-economic deprivation in teenagers and young adults in England. Br J Cancer 96: 1760–1766 17505509 10.1038/sj.bjc.6603794 PMC2359909

[bib3] Birch JM, Pang D, Alston RD, Rowan S, Geraci M, Moran A, Eden TOB (2008) Survival from Cancer in Teenagers and Young Adults in England, 1979. Br J Cancer 99(5): 830–835 18728673 10.1038/sj.bjc.6604460 PMC2528159

[bib4] Bleyer A, Budd T, Montello M (2006) Adolescents and young adults with cancer: the scope of the problem and criticality of clinical trials. Cancer 107: 1645–1655 16906507 10.1002/cncr.22102

[bib5] Bleyer A, Montello M, Budd T, Saxman S (2005) National survival trends of young adults with sarcoma: lack of progress is associated with lack of clinical trial participation. Cancer 103: 1891–1897 15795902 10.1002/cncr.20995

[bib6] Boissel, N, Audere MF, Lheritier V, Perel Y, Thomas X, Leblanc T, Rousselot P, Cayuela JM, Gabert J, Fequeux N, Piquet C, Huguet-Rigal F, Berthou JM, Boiron JM, Pautas C, Michel G, Fiere D, Leverger G, Dombert H, Baruchel A (2003) Should adolescents with ALL be treated as old children or young adults? Comparison of the French FRALL 93 – LALA – 94 Trials. J Clin Oncol 21: 774–780 12610173 10.1200/JCO.2003.02.053

[bib7] Childhood Cancer Research Group (2007) Great Britain Registrations Available at http://www.ccrg.ox.ac.uk/datasets/registrations1975-2000.htm (accessed 29/11/07)

[bib8] De Bont JM, van der Holt B, Dekker AW, van der Does-Van den Berg A, Sonneveld P, Pieters R (2004) Significant difference in outcome for adolescents with ALL treated on pediatric *vs* adult ALL protocols in the Netherlands. Leukaemia 18: 2032–2053 10.1038/sj.leu.2403538 15483674

[bib9] Department of Health (2000) The NHS Cancer Plan: a Plan for Investment, a Plan for Reform'. Crown Copy Right: London

[bib10] Department of Health (2007) Cancer Reform Strategy. Crown Copy Right: London

[bib11] Ferrari A, Bleyer A (2007) Participation of adolescents with cancer in clinical trials. Cancer Treat Rev 7: 603–608 10.1016/j.ctrv.2006.11.005 17250970

[bib12] Geraci M, Birch JM, Alston RD, Moran A, Eden TOB (2007) Cancer mortality in 13–29 year olds in England and Wales 1981–2005. Br J Cancer 97: 1588–1594 17987032 10.1038/sj.bjc.6604080 PMC2360261

[bib13] Hallbook H, Gustafsson G, Smedmyr B, Soderhall S, Heyman M (2006) Treatment outcome in young adults and children >10 years of age with acute lymphoblastic leukaemia in Sweden. Cancer 107: 1551–1561 16955505 10.1002/cncr.22189

[bib14] National Cancer Research Network (2006) Annual Report, 2005/06. National Cancer Research Network: Leeds

[bib15] National Institute for Clinical Excellence (2005) Guidance on Cancer Services -Improving Outcomes in Children and Young people with Cancer. NICE: London

[bib16] O'Brien T, Senner A, Thomas D, Treadgold C, Young A (2006) The need for change, why we need a new model of care for adolescents and young adults with cancer. A Document for Discussion. Presented at Improving the Management of Cancer Services Conference in Melbourne, 2nd March. Available from: URL: http://canteenlive.netx.com.au/documents/AYA-The_Need_for_Change_C7738.ppt (accessed 03/03/08)

[bib17] Office for National Statistics (2005) Cancer Statistics Registrations: Registrations of Cancer Diagnosed in 2004, England. Series MB1 No 32 Office of National Statistics: London

[bib18] Ramanujacher R, Richards S, Hann I, Goldstone A, Mitchell C, Vora A, Rowe J, Webb D (2007) Adolescents with ALL: outcome on UK national paediatric (ALL 97) and adult (UKALL XII/E 2993) trials. Pediatr Blood and Cancer 48: 254–261 10.1002/pbc.20749 16421910

[bib19] Stiller CA, Eatock EM (1999) Patterns of care and survival for children with acute lymphoblastic leukaemia diagnosed between 1980 and 1994. Arch Dis Child 81: 202–208 10451391 10.1136/adc.81.3.202 PMC1718071

[bib20] Stock W, Sather H, Dodge RK, Bloomfield CD, Larson A, Nachman J (2000) Outcome of adolescents and young adults with ALL: a comparison of Children's Cancer Group (CCG) and Cancer and Leukaemia Group B (CALGB) regimens. Blood 96: 467a10887107

